# Supplementary material for: Bayesian parameter estimation for automatic annotation of gene functions using observational data and phylogenetic trees
Source: PLoS Comput Biol. 2021 Feb 18;17(2):e1007948. doi: 10.1371/journal.pcbi.1007948 (PMC7924801; doi:10.1371/journal.pcbi.1007948)
Supplement: Attachment — Submitted filename: response-letter.pdf [file pcbi.1007948.s001.pdf]

Keck School of Medicine of USC

Department of Preventive Medicine

November 3, 2020

RE: Manuscript submission

Dear Editors,

We enclose the revised version of our paper. First, we'd like to apologize for the length of time it has taken us to put this revision together. As noted in the comments, the main critique of the reviewers was the lack of a comparison with existing methods, and in particular with the SIFTER method. As such, we wanted to address this criticism as thoroughly as possible, meaning that it was necessary for us to reanalyze much of our data using SIFTER. As we detail below, this has been an extremely difficult and time-consuming process, and this has been by far the biggest cause for the delay in preparing the revised manuscript. However, we have now completed this process and are therefore able to finalize our revision.

We will begin by detailing our response to this central critique, along with one other "big picture" issue of intent of our work, and follow this with a detailed response to each other reviewer critique. In addition to these changes we have minor textual edits to correct typos etc. Finally, we have edited the abstract and author summary to reflect the changes made below. All changes made are shown in red text in the "changes tracked" version of our revision. We hope that you find our revision responsive, and we look forward to hearing the views of the reviewers.

Many thanks, George Vega Yon, et al.

Main overall criticism

All three reviewers expressed a desire to see our approach benchmarked against existing methods, and in particular the SIFTER method of Engelhardt et al. (2011). As such, we address that critique here.

The central results of our paper show performance of our method on a set of 1,491 annotations over 1,325 genes/proteins. These results are reported in figures 8, 13, and 14. We have added a comparison with SIFTER on these data, as far as proved possible, conducting a leave-one-out cross-validation [LOO-CV] as we did in the analysis with our own method. Overall, we summarize the entire process we conducted as follows:

1. We downloaded SIFTER version 2.1.1 from GitHub and installed it in a large CentOS 7 server with 20 physical cores Intel(R) Xeon(R) CPU E5-2640 v4 @ 2.40GHz and 250 Gb of memory. We downloaded the Gene Ontology [GO] tree and Pfam trees from SIFTER's website, which is dated 2015. The entire installation of SIFTER occupied approximately 150 Gb.

2. Using the EBI/EMBL API, we mapped our genes (“proteins”, for convenience, henceforth) to the corresponding Pfam trees that are available from SIFTER. In many cases, we obtained multiple hits (i.e., trees) per protein. In those cases, we kept and analyzed all hits. This means that for those proteins we obtained more than one prediction when applying SIFTER.

3. In order to run SIFTER using our annotations together with SIFTER's reconciled Pfam trees, it was necessary to write an R wrapper. We will make this R-wrapper available for public use (see below).

4. When running SIFTER we used the option “truncation level” 1 (the default) and 3 (a more complex model), two different runs, and “cross-validation folds equal to zero” to achieve a leave-one-out cross-validation [LOO-CV] that would be directly analogous to that conducted in the results for our own method.

For the analysis in our paper, we focused on proteins for which there were at least two proteins with an annotation of the type “present”, i.e., a “1”, and two others with an annotation of type “absent”, i.e., a “0”, for the same function within the same tree, this is, at least four annotations. These requirements yielded the initial number of ~1,500 annotations over ~1,300 proteins which we used for fitting our evolutionary model. However, when mapping those proteins to Pfam trees, we were not able to use all 1,300 with SIFTER, as many of the resulting trees either reduced to a degenerate tree with a single leaf, or to a tree with a single function, which means that SIFTER cannot be used since it requires at least two functions for inference (if there is just one function, it simply infers all leaves as having that function). Because of these considerations, the initial set of candidate proteins that SIFTER could analyze was reduced from 1,300 to 472.

After running SIFTER for those 472 proteins, the total number of proteins that could be compared with our method further reduced to 147. The reasons for this reflect on a couple of issues that we observed when running SIFTER:

1. In a pre-processing step that SIFTER makes, it prunes the GO tree, which ultimately translates into dropping some annotations. For example, if we passed SIFTER a dataset with 2 GO terms, in some cases it would drop one of those and proceed with the calculations using only a single GO term. This is described in detail in page 18 of the Supplemental Materials of Engelhardt et al. (2011), “[i]n the GO DAG, we first prune all ancestors of nodes with annotations (even if the ancestors themselves have annotations), then we prune all non-annotated nodes. This leaves a set of candidate functions that are neither ancestors nor descendants of each other, ensuring there are no deterministic dependencies between them in terms of the semantic network.”

2. In some other cases, the Pfam version of SIFTER, which is dated 2015, did not include some of the families we identified with the EBI/EMBL API.

3. In another handful of cases, SIFTER was not able to find some of the proteins included in the analysis. Again, similar to the previous point, this was mostly due to the fact that the Pfam database is outdated.

After these cases were excluded, there was only one case in which SIFTER failed to converge and another case in which the final set of proteins was of size one, so leave-one-out cross-validation (LOO-CV) was not possible.

Thus, ultimately, because of the specific requirements of SIFTER, our comparison of methods is conducted on 147 of the ~1300 proteins that we were able to analyze using our own methodology. Nonetheless, this is a reasonable amount of data with which to estimate true- and false-positive rates, so we proceeded with the comparison. The total number of annotations used in this comparison was 184, 18 of which were negative, spread across those 147 proteins.

We calculated mean-absolute-errors (MAEs) and ROC-AUC measurements for each method and reported those in the paper in the new figure 7, which shows the ROC-AUCs curves for both analyses. We found that our method out-performed SIFTER on these data.

We note that this analysis required considerable effort in terms of code-writing and deep-diving into the innards of SIFTER. We are making available to the wider community all the code used to generate our benchmark comparison, together with detailed instructions on how to setup SIFTER and the data for this kind of analysis (which proved to be extremely non-trivial). That code is available on GitHub at https://github.com/USCbiostats/sifter-aphylo, as noted in the revised version of the paper.

These details are now included in the paper in two new subsections between **lines 440-487 and 732-789** respectively.

We note that, during the preparation of the revised manuscript, the number of experimentally annotated trees featured throughout the results section changed. The count reduced from 141 to 138, because it became apparent that three of those families included both negative and positive assertions for a handful of genes, which we were treating as negative assertions only. After excluding those annotations, the three families only had positive assertions, which we could not use for our LOO-CV analysis.

Finally, we would like to mention that conducting this leave-one-out cross-validation (LOO-CV) process using SIFTER for the set of 147 proteins took about 110 minutes, whereas the analysis using our method, aphylo, took about one second.

Big picture issue - intent of paper

While we are happy to include the above benchmarking against SIFTER, and believe it improves the value of the paper, we also wish to note that our goal is not really to compete with, or out-perform the annotation prediction performance of SIFTER at this point. Instead, our paper aims to develop a model in which parameter estimation itself (the precursor to prediction) is feasible not only over a single gene family, but jointly over multiple gene families. We have

attempted to clarify our intent by changing one sentence in the Motivation section of the abstract, and adding the following text to the introduction section of the paper (**lines 51-71**).

"We emphasize that in this paper we focus on developing a model in which parameter estimation is feasible not only over a single gene family, but jointly over multiple gene families. Parameter estimation in SIFTER was attempted for only a single, specific gene family (AMP/adenosine deaminase), and was otherwise infeasible due to multiple factors, including the use of a transition-based function evolution model that results in a combinatorially increasing number of parameters as the number of different functions increases. Further work on SIFTER (Engelhardt et al., 2011) developed an approximation to the likelihood calculation, and did not attempt parameter estimation, instead relying on fixed parameters.

Our goal in this paper is to provide a method that retains computational tractability when estimating the evolutionary parameters, and does not therefore require the use of fixed parameter estimates. Indeed, by adopting a Bayesian perspective we allow the user to allow for uncertainty in parameter estimate by integrating out over the posterior distribution for those parameter values. In order to do so, we simplify the evolutionary model to treat gain and loss of each function independently, minimizing the model parameters. We consider only two properties of each gene family tree: topology and branch type, distinguishing only between branches following speciation versus gene duplication events. Thus, by simplifying the model we regain computational tractability on large trees without paying any great price in terms of prediction error (see Results). This paves the way for future more refined versions of our model that add more realism to the evolutionary model."

Thus, our aim here is to demonstrate that parameter estimation is feasible in such contexts, and additionally yields useful function predictions. We believe the comparison with SIFTER makes this point clearer, and we are grateful to the reviewers for suggesting it, But we have also added a discussion of this question of "goal" to the introduction to make our overall intent clearer. Finally, to emphasize the latter, we have changed the title of the manuscript from "On the automatic annotation of gene functions using observational data and phylogenetic trees", which was rather vague, to "Bayesian parameter estimation for automatic annotation of gene functions using observational data and phylogenetic trees", which more specifically indicates our focus.

We now address the comments of each reviewer in detail:

Reviewer #1

The authors present a computational method for modeling the evolution of gene/protein function annotations in a phylogenetic tree. Since such annotations are typically sparse and noisy, computational approaches for inferring annotations can aid biological and biomedical analyses, and the authors' use of phylogenetic trees for their evolutionary model provides biological justification for the inferred gene functions. The paper is generally clear and well written with appropriate mathematical notation for describing their method, which is easy to understand. However, it would benefit by addressing several questions and concerns.

1. How sensitive is the method to the topology of the phylogenetic trees or missing or spurious edges in the phylogenetic trees?

> *We have added the following discussion of this issue to the Discussion section (see **lines 546-562**):*
>
> *"Sensitivity to misspecified trees. Algorithms such as ours, and that of SIFTER, are built to exploit the information provided by the phylogenetic tree. Loosely speaking, leaves that are most closely related are most likely to have the same function. Whilst it clearly makes sense to exploit this information, it does imply that errors in tree topology may lead to errors in inference of function. In principle, one could extend an algorithm such as ours to explicitly mix over the space of possible trees, but this would have several consequences:*
>
> 1. *We would require an evolutionary model for the topology of the tree itself, rather than just that for evolution of function - this would be a prerequisite for calculating tree-likelihoods, for example.*
> 2. *Computation time would increase by several orders of magnitude.*
>
> *Given these concerns, and our desire to have an approach that retains computational tractability even for large phylogenetic trees (for which the space of possible tree topologies would be enormous), we have not explored this issue in this paper, but approaches in which one mixes over tree space as well have been tried in the coalescent literature for the evolution of individuals within a species, for example in the LAMARC 2.0 package (Mary K. Kuhner, LAMARC 2.0: maximum likelihood and Bayesian estimation of population parameters, Bioinformatics, Volume 22, Issue 6, 15 March 2006, Pages 768–770)."*

2. What is the computational complexity of the method as a function of standard properties (number of nodes, number of edges, depth, etc.) of the phylogenetic tree? The method seems to be very efficient in practice, but it is not clear how well it scales.

> *In practice, the computational complexity of the method reduces to the size of the induced subtree which only includes nodes that can be traced to annotated*

descendants. Felsenstein's pruning algorithm is already linear in the number of leaves, but, because in our model unannotated leaves provide no information, when performing parameter estimation we use a reduced pruning sequence that only includes nodes that can be traced to a descendent with an annotation. The fact that we assume that functions evolve independently also contributes, allowing us to treat trees/proteins with multiple functions as multiple trees, each with one function, instead, computing the likelihoods separately for each combination of tree+function. We have added this and more details as a new subsection on ***lines 161-177****.*

We have also added details for the time taken to perform parameter estimation and the subsequent calculation of posterior probabilities. In our case, the whole parameter estimation took about 3 minutes using MCMC, and calculating the posterior probabilities for all leaves, annotated or not, which involved computing about 83,000 annotations, took about a second. These details have been added to the paper on lines ***301-304***

3. The authors could be more rigorous/precise about how they evaluate their method's performance. Some statements can be difficult to understand, such as "about 73% accuracy using the area under the curve statistic". Some mathematical and statistical terms are used inconsistency or interchangeably, such as accuracy and AUC/AURC and MAE, or are described in non-standard ways. Unfortunately, these issues may be distracting and confusing to some readers.

Thank you for this observation. We were indeed using accuracy and MAE interchangeably and understand that when we used the word accuracy, readers could confuse it with the actual metric of the same name. We now make sure that the word accuracy is used only when its meaning is clear, replacing most instances in which it was used with "MAE", "prediction error", or simply "error" across the manuscript.

4. The method appears to be very robust to missing data and surprisingly seems to result in higher performance with higher missingness. The MAEs of their results also appear to be close to 0.5 for many of the experiments, where 0.5 is what one would expect for a random classifier. It is not clear how well the method is performing or that it is performing as one might expect, e.g., better performance with more data. (Also, the authors say that a "paired t-test found a significant drop in the accuracy levels", but they're not comparing accuracy, the effect size seems very small, and it is unclear if the comparison is overpowered.)

This was a misunderstanding caused by our poor choice of labeling for the relevant figure. The previous caption of ***Figure 2*** *(Figure 1, in the first version of this paper) showing the results of the sensitivity analysis used the word "Accuracy," whereas it should have read "MAE." We have corrected the label, so that now it can be understood that, as the number of available annotations decreases, the Mean Absolute Error increases. We have also changed the caption of the figure to improve clarity:*

"Sensitivity analysis. Boxplots of MAEs as a function of single parameter updates. Sub-figures A1 through D show how the MAEs change as a function of fixing the given parameter value ranging from 0 to 1. In each of these plots, the white box indicates the parameter value used to generate the data (i.e., the ``correct'' parameter value). The last boxplot, sub-figure E, shows the distribution of the MAEs as a function of the amount of available annotation data, that is, how prediction error changes as the degree of missing annotations increases."

*We have changed the statement that says "paired t-test found a significant ..." to "paired t-test found a significant increase in MAE as more data was removed from the model," including a summary table showing the details of the paired t-tests (**lines 367-385**).*

5. Similarly, how does this method compare to similar approaches that use evolutionary models on phylogenetic trees? How does this method compare to other approaches that do not use phylogenetic trees? How does it compare to a very simple baseline approach? Do they have similar MAEs? The authors say that many methods have been developed for inferring gene function, so it would be helpful to compare with some of them. The authors mention SIFTER, but they only compare their method's run times with the projected run times from SIFTER. They do not compare the results or actual run times.

As noted in the opening section of our response letter, we have now added a comparison with SIFTER in order to better benchmark our method. We refer the reader to that response.

6. The authors helpfully perform a detailed sensitivity analysis of their model's parameters. They also apply their method to a number of GO terms and phylogenies from the PANTHER database, but they limit themselves to a small subset (141 of 15,000) of the phylogenies that are relatively well annotated and therefore potentially unrepresentative of what many users may encounter. There could be more discussion of the biological results.

*As described in the opening to this response letter, we have added text to clarify the goals of this paper, which is focused on the issue of parameter estimation and not on large-scale function prediction. As the reviewer points out, our work was focused on the families that have at least two annotations of each type (present and absent), which was necessary in order to assess true positive and true negative rates in a leave-one-out cross-validation. We agree that these trees are generally better-annotated than most, which was one reason we included an analysis of how our performance is affected by missing annotations. We realize that we did not sufficiently discuss this point, and have added text in the sensitivity analysis section highlighting the fact that more needs to be done to understand the behavior of the model under different levels of annotation (**lines 347-355 and 383-385**.)*

Regarding extending the discussion of biological results, we have expanded the Discoveries section to include a comparison of our predictions with predicted annotations from the GO database (predicted by other methods), on ***pages 26-29****, including another figure (****Figure 9****) and table (****Table 7****). We have also identified three mouse proteins for which our predictions are inconsistent with the annotations in the GO database, and briefly discuss these examples.*

Reviewer #2

This paper presents a new phylogeny-based method, *aphylo*, for predicting gene ontology (GO) annotations from data.

The data are gene trees, where each leaf represents a protein sequence that has been annotated based on function (1 = function present and 0 = function absent). Importantly, each gene tree has been **reconciled** based on a rooted species tree, so that the gene tree is rooted and its internal nodes are labeled by evolutionary events (e.g. speciation or duplication). The training data are reconciled gene trees where all leaves have annotations, and the testing data are reconciled gene trees where some but not all leaves have annotations. The goal is then to predict the missing annotations. The proposed method, *aphylo*, achieves this by defining a generic model of function evolution and then utilizing this model within a Bayesian framework.

At a high level, function is modeled as having evolved down the gene tree, meaning that function occurs at the root with some probability and then can be gained or lost on each edge with some probability that depends on whether the node above the edge represents a speciation or duplication event. The posterior distributions of the model parameters (e.g., the probability of gaining function after a gene duplication event) are estimated from the training data within a Bayesian framework (although MLE and MAP estimation is also enabled). Then, at each node, conditioned on these distributions, the posterior probability of function can be computed --- this is used to predict whether function is present (1) or absent (0) at leaves without annotations.

The major weakness of this paper is that *aphylo* (which seems simpler than other phylogeny-based methods for gene ontology annotation) is not benchmarked against other methods (major comment #1).

Because this paper does not evaluate whether *aphylo* improves upon the accuracy or speed of other methods for gene ontology annotation (e.g. those evaluated in the CAFA2 study), it is difficult to evaluate its significance. Therefore, I recommend "Reject".

Major Comments

1. On line 509, the paper reads: "Notwithstanding [*alphylo*'s] simplicity, it provides probabilistic predictions with an accuracy level comparable to that of other more complex, phyla-based models." This is statement is surprising given that the proposed method, *aphylo*, was not benchmarked against other methods (at least I didn't notice this in the main paper). In lines 253-270, there is a brief comparison of *aphylo* to SIFTER [1,2]; however, this discussion is focused on running time not prediction (NOTE: Figures 1-8 in the main text do not seem to show comparisons to SIFTER). Although SIFTER may be too computationally intensive to run on all datasets evaluated in this study, comparisons could be made on at least a small subset of the datasets. If it is impossible to make a comparison to SIFTER, then a comparison could be made to a "base-line method", for example the BLAST-based approach used in the "term-centric evaluation" from the second Critical Assessment of Functional Annotation study (CAFA2) [3].

Term-centric experiments evaluate methods for binary classification tasks, where given a protein sequence, an ontology term (related to molecular function, biological process, cellular component, or human phenotype) is assigned (1 = present and 0 = absent). Several different methods, including SIFTER 2.4 [1,2], were evaluated in CAFA2. Interestingly, Jones-UCL [3] outperformed SIFTER (Fig 7 in [3]); therefore, it seems relevant to compare *aphylo* to Jones-UCL, SIFTER, and a base-line method (assuming that it is computationally feasible to perform such experiments).

> *As noted in the opening section of our response letter, we have now added a comparison with SIFTER in order to better benchmark our method. We refer the reader to that as a response to this observation.*

2. Figure 1 shows the results of several experiments on simulated datasets to evaluate *aphylo* under different conditions (NOTE: Figure 1 would benefit from having the 8 subplots labeled "A", "B", ... "H"). Under ideal conditions (white boxes), the mean MAE is approximately 0.38 (MAE of 0.50 is no better than a random coin toss); however, the MAE takes on a HUGE range. It would be beneficial to do a more fine-grained analysis of datasets with very low and very high MAE. Although justification for showing MAE (instead of ROC curves) is provided, my opinion is that showing ROC curves in addition would be helpful for many readers.

> ***Figure 2** (Figure 1 in the original manuscript) does not show simulated trees, but rather shows how the predictions, and in particular MAEs, of the 138 trees used in the paper change as a function of individual parameter changes. We have added a note clarifying this issue in the paper, and following your advice, added labels to the subfigures to make referencing them easier in the text. See **lines 347-355 and new Figure 2**.*
>
> *In addition, as suggested, we have added the ROC curves corresponding to each estimation method featured in Table 4. See **page 15**.*
>
> *Furthermore, we added an additional figure, **Figure 5 on page 21**, which illustrates a scatter plot of MAEs as a function of number of annotations per tree, including a comparison across the number of negative annotations. See **lines 377-382**.*

3. On line 320, the paper reads: "Finally, we evaluated the impact of removing annotations on accuracy. While a significant drop may not be obvious from the last box in Figure 1, a paired t-test found a significant drop in the accuracy levels." From the description starting on line 536, it sounds like annotations were deleted from gene trees uniformly at random. Does this pattern of missing annotations reflect the pattern of missing annotations in the biological datasets (especially for the lower rates of missing data)? Given the feature example where co-location of missing annotations impacts accuracy, it may be useful to evaluate this issue further (major comment #2).

*We do not address this issue for the simple reason that for the overwhelming majority of proteins, annotations are extremely sparse. The median number of annotations is 12 per tree+go function, and of those only two are of the type "Absent." When annotation is this sparse one cannot really assess the importance of non-random missingness since, even if we try to remove annotations in a way that is clustered, we will very rarely have two annotated leaves sufficiently close together to be affected by this. Thus, we expect results would be essentially identical in the vast majority of cases. We have added a brief discussion of this issue to the paper, explaining this perspective. See **lines 367-376**.*

4. Gene duplication has a huge impact on the probability of gaining function (Tables 3 and 4). Because the gene tree and its reconciliation are estimated from molecular sequence data, it seems important to consider how the accuracy of the estimated gene trees and their reconciliations may impact the accuracy of *aphylo*. Note that gene trees reconciliation may be especially challenging when there is hidden paralogy (https://ecoevorxiv.org/wzcbg/). It would be interesting if strange results, such as parallel evolution of function, ormany gains/losses in function, could be explained by gene tree estimation error or reconciliation error. Along these lines, it also would be helpful to briefly note how gene trees were estimated and reconciled for the PANTHER v11 (as well as if branch support was estimated).

The initial question, about sensitivity to errors in tree topology, was addressed in our response to comment 1. From reviewer 1.

*With regards to the estimation of tree topologies, and their reconciliation in PANTHER, we have added the following text (**lines 248-251**)*

"[W]e used data from the Gene Ontology project, release 2019-02-01 (doi:10.5281/zenodo.2555603) together with PANTHER version 14.1 [Mi et al 2019], which includes about 15,000 reconciled trees reconstructed with the GIGA algorithm [Thomas 2010], modified to include horizontal transfer inference as described in [Mi et al 2016, PMID:26578592]."

Branch support was not estimated, as we do not use this information in our method as implemented here. This decision follows previous work in this field [ref SIFTER], as considering uncertainty in the phylogeny would add substantial complexity to the problem.

Minor Comments

1. Section 3.5 (Discoveries) seems quite valuable, and it may be worthwhile to expand this section and to display the key findings (e.g. for the 10 mouse genes) in a table.

*As suggested, we have expanded this section by adding a table with details regarding the analyzed annotations, including a tabulation comparing our proposed annotations with a Quick GO API search. See **lines 488-525**.*

2. This paper models the evolution of a binary trait (in this case the presence or absence of a particular function), so it may be beneficial to discuss the connections to related work in this area (e.g., [5, 6, 7, 8]).

Thank you for providing those references. Indeed, the phylogenetic regression models have an extensive development on analyzing both binary and continuous traits within the context of non-independent data. But these models use the tree information to build correlation matrices which, conceptually, overlooks our main goal of modelling the evolutionary process itself.

*We went through the referenced papers and added some of them to the discussion and as a reference for other graphical methods for analyzing evolutionary processes (Revell's paper). You can see the additions in **lines 391-393 and 535-540**.*

References

1. B.E. Engelhardt, M.I. Jordan, J.R. Srouji, S.E. Brenner. Genome-scale phylogenetic function annotation of large and diverse protein families. Genome Research 21:1969-1980, 2011. doi: 10.1101/gr.104687.109

2. S.M.E. Sahraeian, K.R. Luo, S.E. Brenner. SIFTER search: a web server for accurate phylogeny-based protein function prediction. Nucleic Acids Research 43:W141-W147, 2015. doi: 10.1093/nar/gkv461

3. Y. Jiang, T. Oron, et al. An expanded evaluation of protein function prediction methods shows an improvement in accuracy. Genome Biology 17:184, 2016. doi: 10.1186/s13059-016-1037-6

4. D. Cozzetto, D. W. Buchan, K. Bryson, and D. T. Jones. Protein function prediction by massive integration of evolutionary analyses and multiple data sources. BMC Bioinformatics 14(Suppl 3):S1, 2013. doi: 10.1186/1471-2105-14-S3-S1

5. A.R. Ives and T. Garland, Jr. Phylogenetic logistic regression for binary dependent variables. Systematic Biology 59:9-26, 2010. doi: 10.1093/sysbio/syp074.

6. L.T. Ho and C. Ane. A Linear-Time Algorithm for Gaussian and Non-Gaussian Trait Evolution Models. Systematic Biology 63:397-408, 2014. doi: 10.1093/sysbio/syu005

Also see https://rdrr.io/cran/phylolm/

7. L.J. Revell. Two new graphical methods for mapping trait evolution on phylogenies. Methods in Ecology and Evolution 4:8, 2013. doi: 10.1111/2041-210X.12066

8. M. Goberna and Miguel Verdu. Predicting microbial traits with phylogenies. The ISME Journal 10:956-967, 2016. doi: 10.1038/ismej.2015.171.

Reviewer #3

The authors present an evolutionary model for gene annotation and use it with a Bayesian MCMC framework to infer unknown gene annotations and even indicate errors in known annotations. This paper is an effort towards automatic gene annotation which is an open and important problem in gene ontology. The paper has some very good features -- the authors explain the model in detail, talk about its drawbacks and possible extensions in the discussion, show the effect of change in prior on the final estimated parameters, show the effect of pooling data and sensitivity analysis and provide a github repository with the code and results. For these reasons I think the paper should be accepted for publication, although I suggest consideration for the following questions and suggestions for the final publication. I have marked the major and minor comments in the list.

1. [Major] The proposed method has not been adequately contrasted with existing methods. For example it would be good to show how the probabilistic framework differs from the one used in SIFTER. Moreover, why have the results not been compared to SIFTER?

> *As noted in the opening section of our response letter, we have now added a comparison with SIFTER in order to better benchmark our method. We refer the reader to that as a response to this observation.*

2. [Major] The sensitivity analysis is a bit confusing. I believe the analysis is done on the PANTHER phylogenies. So if a parameter is shown to have negligible effect on the MAE with other parameter fixed -- is it due to how the model behaves or due to the kind of data that is used in the fitting process? This sensitivity analysis should be repeated on simulated datasets to truly understand which parameters in the model are important for accurate predictions. The paired t-test results mentioned at the end of Section 3.3 should also be shown in the paper.

> *The sensitivity analysis shown was performed as a way to illustrate the sorts of applications that are possible by a computationally tractable method as ours. We have clarified the description of that study to improve readability. Clearly, such a study could also be extended, but given the other changes made in this revision, including the addition of a comparison with SIFTER, our paper is already a rather long one. The data we used for the existing exemplar analysis is reasonably large (about 1,300 proteins) and we feel gives a good indication of the sorts of reuts that might be obtained in a wider study of this issue, which might be performed in future work.*
>
> *We have updated most of the sensitivity analysis section to reflect these points. See* ***lines 347-355 and 367-385****.*

3. [Minor] What are the system specifications for the run-time analysis between the proposed method and SIFTER at the end of page 12? `Regular laptop computer' is not informative enough. Please provide the processor used, its speed and RAM for the system.

> *We have added a new subsection in Materials and Methods detailing the hardware used. See **lines 790-797**.*

4. [Minor] The discovery section looks at the solutions with high certainty in prediction of presence or absence of function.On the other hand Featured Examples section presents one low accuracy result that reveals an area of inconsistency in current literature. It would be helpful to describe other putative areas where the current knowledge might be inconsistent based on the predictions of your method.

> *Our focus in this paper is on methods development for, and presentation of, a computationally tractable method for parameter estimation and annotation inference. As such, we intended the discovery section as an exemplar analysis of the sorts of things that become possible with such an algorithm. We have expanded the Discoveries section to include a comparison of our predictions with predicted annotations from the GO database (predicted by other methods), on **pages 26-29**, including another figure (Figure 9) and table (Table 7). We have also identified three mouse proteins for which our predictions are inconsistent with the annotations in the GO database, and briefly discuss these examples.*
