# Supplementary material for: Bayesian parameter estimation for automatic annotation of gene functions using observational data and phylogenetic trees
Source: PLoS Comput Biol. 2021 Feb 18;17(2):e1007948. doi: 10.1371/journal.pcbi.1007948 (PMC7924801; doi:10.1371/journal.pcbi.1007948)
Supplement: Attachment — Submitted filename: response-letter.pdf [file pcbi.1007948.s002.pdf]

Keck School of Medicine of USC

Department of Preventive Medicine

December 14, 2020

RE: Second Revision aphylo paper

Dear Editors,

Thank you very much for the quick second revision of our manuscript; we much appreciate it. Please find enclosed our revised manuscript addressing the issues indicated by Reviewer 1. Changes have been highlighted in red.

Sincerely,

George G. Vega Yon, et al.

Reviewer #1 comments:

The authors made good-faith efforts to address the reviewer comments, and they have largely addressed my concerns. I have a few comments about their resubmission:

1. Each reviewer requested that the authors compare aphylo with existing methods, so the authors compared aphylo with SIFTER. This comparison required a substantial amount of effort, and the authors have helpfully shared their code publicly. However, the authors also noted that the comparison is somewhat ***awkward because aphylo and SIFTER have different objectives****. I think that there is still an opportunity for more relevant benchmarking and for better clarifying aphylo's place in the computational landscape of phylogenetic approaches for gene function annotation. For example, should readers use aphylo instead of other methods in their current pipelines, should they use aphylo to choose parameters for other methods, etc.?*

Our response:

> We have added the following text to the Discussion (**lines 536-548**) to provide guidelines that address this point:
>
> "Regarding the question of when one should use aphylo versus SIFTER, or other methods, we note that if both negative and positive assertions are available, aphylo should perhaps be the method of choice since it explicitly uses both types of annotations which, as suggested in our analyses, should provide more accurate predictions than SIFTER. However, when only positive annotations are available, parameter estimation becomes problematic as estimates will tend to reflect a tree with positive assertions only. Unless one is willing to place strong priors on parameter values, parameter estimation under this scenario is not recommended. That said, for making predictions using parameters previously obtained from another training set (e.g. those in this paper), using aphylo is possible even when only positive annotations are available. Using pre-set parameters, the efficiency of aphylo would allow it to be incorporated into existing data-analysis pipelines to obtain a "second opinion" alongside other prediction methods, at a relatively little computational cost."

2. The authors say that users can perform large-scale analysis with a "regular" computer, but they later describe a computer with 20 CPU cores and 250 GB RAM. Are these the same or different computers? Can the authors unambiguously describe the system or system requirements for aphylo on the analysis in the paper?

Our response:

> To clarify this issue, we have changed the text in two places. First, the paragraph that formerly read:

“From the practitioner's point of view, users can perform large scale analyses, including both parameter estimation and subsequent function prediction, using no more than a ``regular'' computer.”

Has changed to (**lines 172-173**):

“From the practitioner's point of view, users can perform large scale analyses, including both parameter estimation and subsequent function prediction, using a single computational thread and less than one Gb of RAM, i.e., no more than a ``regular'' computer.”

Second, in the original version of the paper the analysis that involved ~1,300 genes, which included both parameter estimation and prediction, was conducted using a laptop computer with 8Gb of RAM on a single thread. The large server we mentioned in the paper was used for the revised manuscript to handle SIFTER.

The complete installation of SIFTER required about 150 Gb of RAM, which is why we introduced the large server mentioned in the technical specifications. To make the comparison between aphylo and SIFTER the fairest possible, we re-ran the aphylo analysis on that same server. We now clarify this in the following new text (**lines 811-812**):

“The analyses using GO annotations, involving the 1,300 experimentally annotated proteins were run on a large CentOS 7 server with 20 physical cores Intel(R) Xeon(R) CPU E5-2640 v4 @ 2.40GHz and 250 Gb of memory. Nevertheless, all calculations involving the 1,300 proteins were performed using a single computational thread **and less than one GB of memory.**”

3. It was not immediately clear what was changed and what was kept from the previous version of the manuscript because there were no highlights, etc. However, there are several opportunities to clean up the submission, particularly in the figures and figure lengths. For example, the y-axis for Figure 10 is "Value", and the y-axis for Figure 13 is "aphylo - SIFTER", so the authors could be more precise.

Our response:

We have edited the axis labels to make them clearer on both those figures, as well as making one or two minor changes to figure captions to improve clarity in **figures 10 and 13**.
